# Random Thoughts

**Published:** 1859-10

**Authors:** Geo. Watt


### RANDOM THOUGHTS.

BY GEO. WATT.

THIS paper is what its name imports. The thoughts here recorded are set down without any regard to order. Many of them were noted, in memorandum, on waste paper, by the wayside, or at public meetings when the proceedings proved uninteresting. Some were suggested by the casual remark of a professional brother, some by a glance at a sentence in an article which time did not permit us to read; and some were not suggested at all, but sprung into existence of their own accord. We write them down to be relieved of them, just as a young lady marries an importunate lover, "to get quit of him."

PROFESSIONAL HOBBIES.—This term is generally used in an opprobrious sense—but why? Does the traveler treat with contempt, and speak disparagingly of the faithful courser that has carried him safely to the end of a successful journey? And, especially if he is traveling for a prize, and his steed has carried him far in advance of all competitors, is he not tender in his caresses, and faithful in his attentions to the noble animal? And if the prize can only be gained by making the journey, and the way is so long and so difficult that but few animals can endure the travel, would we not regard as an ungrateful wretch, the successful rider, should he malign or maltreat the faithful companion that carried him to the goal? And what if those who are distanced in the race disparage the qualities of his steed? Will he therefore discard him and go afoot, ride a donkey, or a spavined hack, or abandon the race entirely?

Well, to bring this thought home, the whole dental profession is, or should be on a journey. The way is long and difficult. It is rough, uneven, and steep. It leads from the depths of professional ignorance and degradation to the hights of professional knowledge and respect. A prize is held out to all who make the journey and reach the goal—a prize made up of a series of prizes—which consists of all those blessings and enjoyments which make the life of the true professional man desirable over that of the charlatan and the impostor.

In a race or a journey, those who ride usually advance faster than those who walk. And, if the pathway be unexplored, those in the rear are dependent on those in advance for a knowledge of the way, and for the information which is necessary to overcome its difficulties. So in our profession, those who ride are in the advance, and those who walk follow behind; while those who stand still, or sit idle, are left out of sight, and cease to be regarded as members of the *body professional.*

But what do those in advance ride on? On HOBBIES—*professional hobbies;* for there are no railroads to knowledge in the dental profession. And why not ride hobbies, and rejoice in the qualities and capacities of our steeds? What is a hobby? Compare Webster's 1st and 3d definitions: "A strong, active horse;"—"That which a person pursues with zeal or delight." No mere pony or donkey, but a good strong courser which we may ride with "delight."

A professional hobby rider, then, is one who selects some subject or professional principle as his favorite, examines it in all its bearings, pursues it in all its ramifications, and makes it, throughout, tributary to the wants of his profession. And his researches in this direction are a success, like that of the traveler who, pressing in one direction, surveys a route or explores a river, while he who tries to travel two ways at once, fails to make progress in any direction.

All professional advancement has been and is made by hobby riding; but many fail to ride to the best advantage. Some mount a hobby, but finding it difficult to ride, dismount, and take a fresh one. This is soon deserted, and another is taken up,—each one being left before it is broken into rank, and, of course, but little progress is made. Others, again, mount their favorites, with whip and spur, and ride them to death, without reaching their journey's end; and, being themselves too far exhausted to arrest a fresh steed, pine by the way, while others pass them, and reach the goal.

Our advice is to select some favorite subject, pursue it with zeal and push it with energy; but stop and rest before you are too much engrossed by it to think impartially on other subjects, and especially before the profession has become weary of hearing it. But don't cease to make progress. Lay hold of another, while this one rests, and follow it up with equal energy; and don't get discouraged if you are charged with riding hobbies.

PLATINUM.—We have no notion of writing a chemical dissertation on this "noble" metal. We only wish to retrieve its name from the gross profanations to which it is subject in our profession. A *noble* metal has the same right to its title that a *noble*-man has. A chubby little boy, as he trundles his hoop, may with propriety be called "Jim along Josey;" but, when he goes with "a satchel to school," he reports his name as James A. Joseph; and, in due time, may be entitled the "Hon. J. Along Joseph, Esq."

Well, this metal was at first called "platina," a diminutive of *plata*, silver. Being found in minute quantities, and having the color of silver, it was given a name which implied "little silver."

When it became better known, it was named in accordance with the approved system of nomenclature, and called

PLATINUM. Let us drop the nickname, adopt the true one, and write it platinum. But if we must retain the old orthography, let us not pronounce it pla-*teen*-a, but plat-i-na, accenting the first syllable.

This is a small matter; but the credit of the professian is somewhat at stake. At the late Convention almost every member who had occasion to speak of the metal, used the obsolete, barbarous pronunciation, giving to strangers the impression that we were lacking both in taste and science.

ULCERATION.—This term is very vaguely used by a majority of our profession. If alveolar abscess is referred to, it is often said that ulceration has taken place. In a large majority of the cases in which the term is used, *suppuration*, and not ulceration is meant. If we pervert the word thus we must needs coin a new one to express the true meaning of ulceration. Many cases reported in the journals as well as in verbal discussions, are unintelligible, on account of the vagueness with which this and similar words are used.

(TO BE CONTINUED.)

